# Human Spermatogenesis Tolerates Massive Size Reduction of the Pseudoautosomal Region

**DOI:** 10.1093/gbe/evaa168

**Published:** 2020-08-12

**Authors:** Maki Fukami, Yasuko Fujisawa, Hiroyuki Ono, Tomoko Jinno, Tsutomu Ogata

**Affiliations:** e1 Department of Molecular Endocrinology, National Research Institute for Child Health and Development, Tokyo, Japan; e2 Department of Pediatrics, Hamamatsu University School of Medicine, Japan

**Keywords:** crossover, homologous recombination, pseudoautosomal region, sex chromosome, SHOX

## Abstract

Mammalian male meiosis requires homologous recombination between the X and Y chromosomes. In humans, such recombination occurs exclusively in the short arm pseudoautosomal region (PAR1) of 2.699 Mb in size. Although it is known that complete deletion of PAR1 causes spermatogenic arrest, no studies have addressed to what extent male meiosis tolerates PAR1 size reduction. Here, we report two families in which PAR1 partial deletions were transmitted from fathers to their offspring. Cytogenetic analyses revealed that a ∼400-kb segment at the centromeric end of PAR1, which accounts for only 14.8% of normal PAR1 and 0.26% and 0.68% of the X and Y chromosomes, respectively, is sufficient to mediate sex chromosomal recombination during spermatogenesis. These results highlight the extreme recombinogenic activity of human PAR1. Our data, in conjunction with previous findings from animal studies, indicate that the minimal size requirement of mammalian PARs to maintain male fertility is fairly small.

**Significance**

Mammalian X and Y chromosomes consist of different genetic components, except for small homologous chromosomal elements designated as pseudoautosomal regions (PARs). PARs are essential for spermatogenesis, because they serve as the sole platform for X–Y crossovers during meiosis. Here, we show that loss of 85% of human PAR does not necessarily result in male infertility, based on the observation of two men who carried chromosomal deletions within PAR. Our findings suggest that mammalian PARs have a potential to acquire gross structural changes during evolution without affecting male reproductive fitness.

## Introduction

Homologous recombination between the X and Y chromosomes is indispensable for mammalian male meiosis (Koller and Darlington 1934; Simmler et al. 1985; Raudsepp and Chowdhary 2015). Such recombination occurs exclusively in the pseudoautosomal regions (PARs), in which the two sex chromosomes share homologous sequences (Simmler et al. 1985; Skaletsky et al. 2003; Ross et al. 2005). Although the human genome contains two PARs, only PAR1 spanning 2.699 Mb at the end of Xp/Yp (GRCh37/hg19) serves as the platform for sex chromosomal recombination (Charchar et al. 2003; Raudsepp and Chowdhary 2015).

The high frequency of meiotic recombination in PAR1 gives rise to various deletions and duplications (Bussell et al. 2006; Otto et al. 2011; Shima et al. 2016). Of note, Gabriel-Robez et al. (1990) and Mohandas et al. (1992) identified Xp terminal deletions encompassing the entire PAR1 in two men with spermatogenic arrest. These X chromosomal deletions contained no known spermatogenic genes, indicating that infertility of these two men results from the loss of PAR1. Consistent with this, animal studies revealed that the structural or sequence divergences of PARs between two murine subspecies affect the success rate of X–Y crossover (Dumont 2017). To date, however, no study has addressed to what extent human spermatogenesis tolerates PAR1 size reduction.

## Results and Discussion

**Fig. 1:**
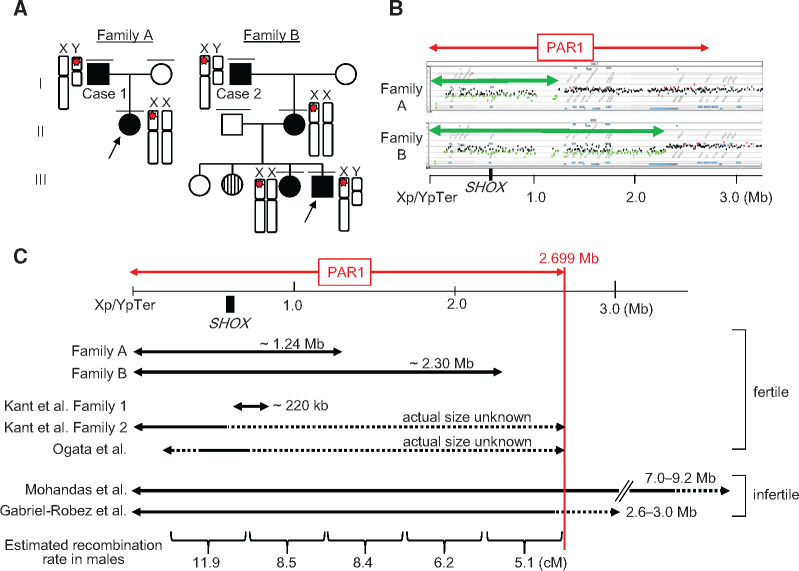
Molecular findings of families A and B. (*A*) The pedigrees of families A and B. Black boxes and circles indicate individuals with mesomelic short stature and/or skeletal deformities, whereas the white box and circles depict unaffected family members. The striped circle indicates an individual with short stature, whose genomic DNA sample and detailed clinical information were unavailable. Red stars on the X and Y chromosomes indicate *SHOX*-containing deletions in the pseudoautosomal region 1 (PAR1). (*B*) Representative results of microarray-based comparative genomic hybridization for the probands of families A and B. PAR1 is indicated by the red arrow. Black, green, and red dots denote signals indicative of the normal, decreased (<−0.8) and increased (>+0.4) copy numbers, respectively. Green arrows indicate the deleted regions in families A and B. Genomic positions refer to the human genome database (GRCh37/hg19). The position of *SHOX* is indicated by the black box. (*C*) Schematic representation of PAR1. The deleted regions in families A and B, together with those in the three previously reported cases with normal fertility (Ogata et al. 2002; Kant et al. 2011) and two cases with spermatogenic failure (Gabriel-Robez et al. 1990; Mohandas et al. 1992), are shown as black arrows. The broken lines depict dosage-unknown regions. The position of *SHOX* is indicated by the black box. The panel at the bottom shows the recombination rates of normal males (in cM) calculated by Hinch et al. (2014).

We identified two families (families A and B) with large terminal deletions of PAR1 (fig. 1*A*). These families were found through molecular analyses of *SHOX*, an osteogenic gene located in PAR1 at a position ∼600 kb from the end of Xp/Yp (Rao et al. 1997; the UCSC Genome Browser, http://genome.ucsc.edu/ [GRCh37/hg19]). The probands of these families (II-1 of family A and III-4 of family B) presented with mesomelic short stature and skeletal deformity indicative of *SHOX* haploinsufficiency (Belin et al. 1998). Multiplex ligation-dependent probe amplification showed decreased copy number of all *SHOX* exons and their flanking regions in both individuals. The same copy-number losses were also identified in their relatives (fig. 1*A*). Microarray-based comparative genomic hybridization (CGH) detected PAR1 terminal deletions of ∼1.24 Mb (maximum interval, chrXY:1–1,256,608; minimum interval, chrXY:1–1,235,344) in family A and of ∼2.30 Mb (maximum interval, chrXY:1–2,309,402; minimum interval, chrXY:1–2,297,925) in family B (fig. 1*B*). Fluorescent in situ hybridization revealed that the deletion in family A was located on the X chromosome of the proband and on the Y chromosome of her father (A-I-1) (supplementary fig. 1, Supplementary Material online), whereas the deletion in family B resided on the X chromosome of the proband, his elder sister (B-II-3), mother (B-II-2), and maternal grandfather (B-I-1) (fig. 1*A*). All deletion-positive individuals exhibited skeletal features indicative of *SHOX* haploinsufficiency (Belin et al. 1998), but no other congenital anomalies. Allegedly, another individual of family B (III-2) also had short stature, although genomic DNA samples and detailed clinical information of this individual were unavailable.

The most striking finding from these families was that two adult men, that is, the proband’s father in family A (A-I-1; hereafter referred to as case 1) and the proband’s grandfather in family B (B-I-1; case 2), were fertile and transmitted their PAR1 deletions to daughters (fig. 1*A*). Cases 1 and 2 retained only ∼1.44-Mb and ∼400-kb segments of PAR1, respectively (fig. 1*B*). In case 1, homologous recombination between the X and Y chromosomes must have occurred within the ∼1.44-Mb segment in the most centromeric part of PAR1, because during meiosis, the *SHOX*-containing deletion was translocated from the Y chromosome to the X chromosome (fig. 1*A* and supplementary fig. 1, Supplementary Material online). The normal female phenotype of the daughter of case 1 (A-II-1) provides evidence that the X–Y crossover in case 1 occurred telomeric to *SRY*, the sex-determining gene located in the Y-specific region only ∼5 kb from the PAR1 boundary (the UCSC Genome Browser). It is known that male meiotic homologous recombination occurs predominantly in the telomeric part of PAR1, with the hottest hotspot being at the *SHOX* locus (May et al. 2002; Flaquer et al. 2009; Hinch et al. 2014). Moreover, in several species, telomeric regions are predicted to play an important role in the meiotic chromosomal pairing (McKee 2004). However, the results of case 1 indicate that loss of the telomeric half of PAR1 does not necessarily lead to spermatogenic failure. Consistent with this, previous studies have identified three fertile men with PAR1 partial deletions, in whom meiotic homologous recombination occurred between *SHOX* and the centromeric end of PAR1 (fig. 1*C*) (Ogata et al. 2002; Kant et al. 2011). In case 2, furthermore, the site of meiotic recombination was restricted to a ∼400-kb region at the most centromeric part of PAR1. The *SHOX*-containing deletion in this individual resided on the X chromosome throughout meiosis, indicating that the recombination occurred between the Y chromosome and the nontransmitted sister chromatid of the X chromosome. We cannot completely exclude the possibility that the sex chromosomal recombination in case 2 occurred outside PAR1. For example, PAR2 on Xq/Yq also has the potential to mediate male meiotic recombination (Ciccodicola et al. 2000; Raudsepp and Chowdhary 2015). However, this probability is low, because 1) complete loss of X chromosomal PAR1 was observed in two men with spermatogenetic arrest (Gabriel-Robez et al. 1990; Mohandas et al. 1992), 2) the estimated genetic size of PAR1 in normal males is ∼50 cM (Flaquer et al. 2009; Evers et al. 2011; Otto et al. 2011), suggesting that virtually all spermatocytes leading to live births undergo homologous recombination in this region, and 3) in midpachytene spermatocytes, chiasmata were observed exclusively in PAR1 (Sarbajna et al. 2012). Of note, the ∼400-kb PAR1 segment retained in case 2 accounts for only 14.8% of normal PAR1 and corresponds to 0.26% and 0.68% of the length of the X and Y chromosomes, respectively (the UCSC Genome Browser). The estimated genetic size of this segment in normal males is <5 cM (fig. 1*C*; Hinch et al. 2014), indicating that during normal spermatogenesis, this short segment is rarely involved in sex chromosomal recombination. Nevertheless, in case 2, this segment is likely to have hosted homologous recombination in most spermatocytes, because animal studies have shown that X–Y pairing in 50% of germ cells, but not in 30% of cells, permits sperm production (Faisal and Kauppi 2016).

The aforementioned results indicate that the minimal size requirement of human PAR1 to maintain spermatogenesis is fairly small. In this regard, it is noteworthy that the size of PARs is highly variable among mammalian species (Graves et al. 1998; Raudsepp and Chowdhary 2015). PARs are believed to be under the constant evolutionary pressure to shrink, yet such PAR attrition can be counteracted by the insertion of DNA fragments through chromosomal translocation (Graves et al. 1998; Mensah et al. 2014). Indeed, recent studies have shown that a small percentage of healthy men carry a ∼110-kb insertion polymorphism in PAR1 that expands the size of the recombination platform to some extent (Mensah et al. 2014; Poriswanish et al. 2018). Thus, human PAR1 is still evolving. The present study provides evidence that human PAR1 is highly tolerant to size reduction. These data are consistent with the prior observation that the size of murine PARs is only 700 kb or less (Perry et al. 2001; Raudsepp and Chowdhary 2015). The high recombinogenic activity of mammalian PARs is likely to reflect their long chromosome axes, which leads to the frequent occurrence of double-strand DNA breaks (Kauppi et al. 2011; Acquaviva et al. 2020).

In summary, the results indicate that a ∼400-kb segment at the centromeric end of PAR1 is sufficient to produce homologous recombination during human spermatogenesis. This study highlights the extreme recombinogenic activity of PARs in the maintenance of male fertility.

## Materials and Methods

This study was approved by the Institutional Review Board Committee and performed after obtaining written informed consent to participate. The probands of families A and B were identified through molecular analyses of *SHOX* for patients with short stature and/or skeletal deformity. The other members of these families were ascertained by familial studies of the probands.

Genomic DNA samples were obtained from peripheral leukocytes of the participants. Multiplex ligation-dependent probe amplification for *SHOX* and its flanking regions was performed using the commercially available kit (SALSA P018-G1, MRC-Holland, the Netherlands). CGH was performed using a human catalog array (4×  180 k format; Agilent Technologies, California, USA) according to the manufacturer’s instructions. The results of CGH were assessed using the Genomic Workbench (version 7.0, Agilent Technologies) with the default settings of the aberration detection algorithm.

Metaphase spreads were generated from peripheral leukocytes. Fluorescent in situ hybridization was carried out using a standard procedure (LSI Medience, Tokyo, Japan). We utilized a ∼14-kb probe containing *SHOX* exons 3–5 and a part of exon 6a and a probe for the Xq/Yq telomere region (TelVysion VYS33-260023; Abbott Laboratories, Illinois, USA). The cells were stained with 4′,6-diamidino-2-phenylindole, dihydrochloride (ThermoFisher Scientific, Massachusetts, USA).

## Supplementary Material

Supplementary data are available at *Genome Biology and Evolution* online.

## Supplementary Material

evaa168_Supplementary_Data
