# Supplementary material for: Human Spermatogenesis Tolerates Massive Size Reduction of the Pseudoautosomal Region
Source: Genome Biol Evol. 2020 Aug 12;12(11):1961–4. doi: 10.1093/gbe/evaa168 (PMC7608489; doi:10.1093/gbe/evaa168)
Supplement: evaa168_Supplementary_Data [file evaa168_supplementary_data.pdf]

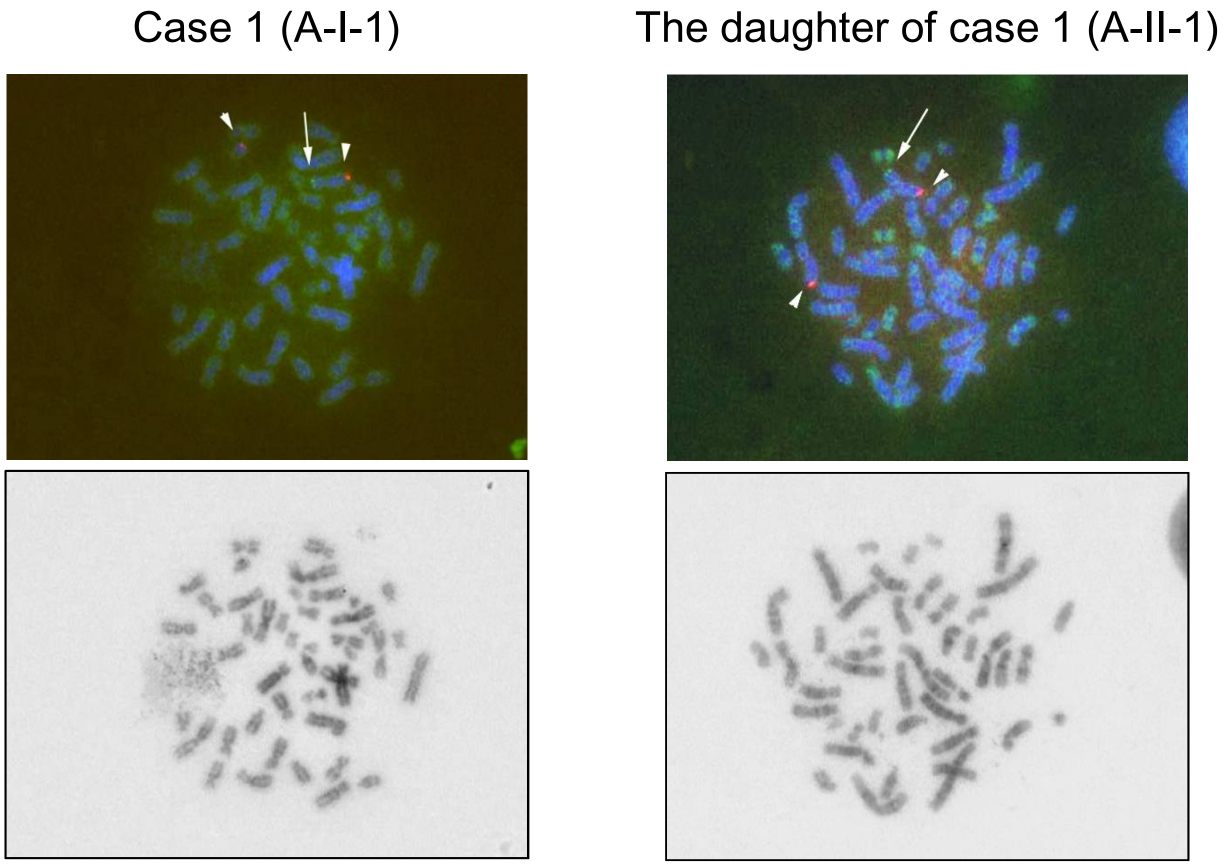


**Supplementary fig. 1.** Fluorescent *in situ* hybridization of family A.
White arrows depict *SHOX* (green signals), and white arrowheads indicate the Xq/Yq telomere region (red signals). The *SHOX*-containing PAR1 deletion was located on the Y chromosome of case 1 (A-I-1) and on the X chromosome of his daughter (A-II-1). The lower panel shows the results of 4',6-diamidino-2-phenylindole, dihydrochloride (DAPI) staining.
